# Brainstem Toxicity Following Proton Beam Radiation Therapy in Pediatric Brain Tumors: A Systematic Review and Meta-Analysis

**DOI:** 10.3390/cancers16213655

**Published:** 2024-10-30

**Authors:** Abdulrahim Saleh Alrasheed, Abdulsalam Mohammed Aleid, Reema Ahmed Alharbi, Mostafa Habeeb Alhodibi, Abdulmonem Ali Alhussain, Awn Abdulmohsen Alessa, Sami Fadhel Almalki

**Affiliations:** 1 Department of Neurosurgery, College of Medicine, King Faisal University, Alahsa 31982, Saudi Arabia; 221414880@student.kfu.edu.sa; 2 Department of Surgery, College of Medicine, King Faisal University, Alahsa 31982, Saudi Arabia; abdulsalamaleid@abjad.bio; 3 Department of Neurosurgery, Faculty of Medicine, University of Tabuk, Tabuk 31982, Saudi Arabia; 391006970@stu.ut.edu.sa; 4 Department of Family Medicine, Alfudhool Primary Healthcare, Al-Ahsa Health Cluster, Alahsa 31982, Saudi Arabia; malhodibi@moh.gov.sa; 5 Department of Neurosurgery, King Fahad Hospital, Hofuf 36441, Saudi Arabia; abalalhussain@moh.gov.sa (A.A.A.); awabalessa@moh.gov.sa (A.A.A.)

**Keywords:** brain tumors, proton beam radiation therapy, brainstem toxicity, brainstem injury, brainstem necrosis, pediatrics

## Abstract

**Background:** Proton beam radiation therapy (PBRT) is an advanced cancer treatment modality that utilizes the distinctive physical properties of protons to precisely deliver radiation to tumor targets while sparing healthy tissue. This cannot be obtained with photon radiation. In this systematic review and meta-analysis, we aimed to comprehensively assess the risk of brainstem toxicity in pediatric brain tumor patients undergoing PBRT. **Methods:** With adherence to the Preferred Reporting Items for Systematic Reviews and Meta-Analyses (PRISMA) guidelines, a predetermined search strategy was used to identify eligible articles from PubMed, Web of Science, Scopus, and Cochrane Library through July 2024. **Results:** The current study included a total of 11 eligible articles. The pooled prevalence of patients who suffered from brainstem toxicity was 1.8% (95% CI: 1%, 2.6%). The pooled prevalences of patients with Grade 1 to Grade 5 brainstem toxicity were found to be 10.6% (95% CI: 8.8%, 30%), 1.5% (95% CI: 0.6%, 2.5%), 0.7% (95% CI: 0.3%, 1.1%), 0.4% (95% CI: 0.1%, 0.7%), and 0.4% (95% CI: 0.1%, 0.8%), respectively, with an overall pooled prevalence of 0.7% (95% CI: 0.4%, 1%). **Conclusions:** This study revealed a relatively low incidence of symptomatic brainstem toxicity and its related mortality in the pediatric population undergoing PBRT. However, further research is encouraged to study the broader effects of PBRT and to explore various factors that may influence the risk of brainstem toxicity in patients treated with PBRT.

## Simple Summary

Proton beam radiation therapy is one of the major treatment modalities used for cancer treatment, including brain tumors. This treatment modality is distinct from other radiation options because of its ability to deliver radiation to tumor targets while sparing healthy tissue. Brainstem toxicity is a rare but important complication can arise due to exposure to radiation; hence, in this systematic review and meta-analysis, we aimed to explore the risk of brainstem toxicity in pediatric brain tumor patients undergoing proton beam radiation, focusing on quantifying its incidence and severity. Eleven articles were considered eligible in our study, and the results yielded an overall brainstem toxicity incidence of 1.8%, ranging in severity, with Grade 1 brainstem toxicity (asymptomatic) being the most common. This study revealed a low incidence of symptomatic brainstem toxicity and related mortality among pediatric brain tumor patients undergoing proton beam radiation, which could support the idea of it having a good toxicity profile and possibly re-enforces the need for more comprehensive primary studies regarding this radiation modality in brain tumor patients to uncover unknowns and help us understand the grey areas of this topic.

## 1. Introduction

Pediatric brain tumors are the most common solid tumors found in children and encompass a diverse range of forms. Using contemporary therapeutic approaches, the average 5-year survival rate is approximately 75% [1]. Radiation therapy is an important part of curative treatment, but it can have serious side effects, including neurocognitive deficits, neuroendocrine abnormalities, damage to blood vessels, loss of hearing, permanent hair loss, and an increased risk of developing other types of cancer due to the exposure of nearby healthy tissue [2,3]. Proton beam radiation therapy (PBRT) minimizes radiation exposure to healthy tissues while effectively targeting the tumor. The preservation of normal tissue in proton treatment is made possible through the distinctive physical properties of protons, which enable precise control over radiation delivery at a certain depth—a level of control that cannot be achieved with photon therapy [4,5].

Although PBRT has clear benefits compared to photon therapy for pediatric cranial radiation, the occurrence of brainstem necrosis in some cases over the last decade has raised concerns about a potentially distinct risk profile of necrosis, which is different from the low risk observed with photon therapy [6,7]. The National Cancer Institute (NCI) Workshop on PBRT for Children was held specifically to address the risk of brainstem injury after PBRT and the dosimetric parameters required to prevent it. Despite the low risk of brainstem injury, its association with significant morbidity necessitates the prevention of such a risk. Strict implementation and adherence to brainstem dosimetric parameters have been shown to be effective in minimizing the risk of brainstem necrosis [8,9].

There are concerns over the spatial arrangement of energy deposition events triggered by protons and the intricate radiation-induced damage that can result from this [10]. The average relative biological effectiveness (RBE) of protons compared to photons is 1.1 for standard fractionation. However, the RBE may vary depending on the location and can increase to 1.3 or 1.4 at the distal end of the Bragg peak [11,12]. Additionally, the occurrence of brainstem injury, albeit rare, has been documented in early research. This has led to apprehension regarding the application of PBRT for malignancies located in the posterior fossa. Researchers at St. Jude Children’s Research Hospital (SJCRH) discovered that brainstem necrosis occurred in 3.7% of patients within five years after receiving photon-based radiation. The prevalence ranged from 2.2% to 8.6% in other trials including children [13,14,15]. Research assessing the likelihood of brainstem injury caused by protons has determined a wider spectrum of risk, ranging from 0.7% to 16% [6,16,17]. This is mostly due to the presence of a diverse patient group and the absence of a universally agreed-upon definition of brainstem damage. The causality of brainstem injury is influenced by multiple factors, including the dose, volume, and technique of radiotherapy; the age of the patient at the time of treatment; the location of the tumor in the infratentorial region; the amount and number of surgical resections; and the use of high-dose chemotherapy with stem cell rescue, among other factors [9,18]. In the present systematic review and meta-analysis, we aimed to provide an overview of the risk of brainstem toxicity in the pediatric population undergoing PBRT compared to other radiation modalities when feasible.

## 2. Methods

This systematic review and meta-analysis was conducted according to the recommendations of the Cochrane Handbook of Systematic Reviews of Interventions [19], with the adherence to the Preferred Reporting Items for Systematic Reviews and Meta-Analyses (PRISMA) guidelines [20]. A prospective protocol was registered in the International Prospective Register of Systematic Reviews (PROSPERO) (registration number: CRD42024563845).

### 2.1. Database Searching

We conducted a comprehensive literature search of PubMed, Web of Science, Scopus, and Cochrane using the following search strategy: ((Brain stem toxicity) OR (“Brain Stem” [Mesh] OR “brain stem”) AND (“Toxicity” [Mesh] OR “toxicity” OR “adverse effects” OR “side effects”)) AND (“Proton Therapy” [Mesh] OR “proton beam radiation” OR “proton beam therapy” OR “proton beam”). Studies published from inception until July 2024 were considered eligible for the screening process

### 2.2. Screening

EndNote version 7 software [21] was used to remove duplicates from the literature search results, after which the remaining articles were imported into Rayyan version 5.2 software [22] to facilitate the screening process. Two independent authors evaluated the titles and abstracts based on the inclusion criteria to determine potentially eligible articles. Studies that complied with the inclusion criteria were then evaluated based on their full text by two independent reviewers to confirm their eligibility. Any conflicts that arose during the screening process were resolved either by consensus or referral to a senior author for resolution.

### 2.3. Inclusion and Exclusion Criteria

English observational studies (cohort, cross-sectional, or case–control) and randomized controlled trials (RCTs) investigating the effect of PBRT on brainstem toxicity in pediatric brain tumors were included. Studies not reporting the outcome of interest were excluded.

### 2.4. Quality Assessment

Two independent authors conducted the quality and risk of bias assessment for the included observational cohort studies using the Newcastle–Ottawa scale (NOS) tool. Any discrepancies that arose during this process were resolved by consulting a third reviewer. The tool consists of eight questions, with a maximum of one star for each, except for the comparability question, which can receive two stars. Therefore, the highest score is nine and the lowest score is zero. Studies scoring from 0 to 3 were considered of low quality, 4 to 6 were of moderate quality, and 7 to 9 were of high quality [23].

### 2.5. Data Extraction

Using Microsoft Excel sheets, two independent authors reviewed the full text of the included studies to extract the variables of interest, including baseline data (study design, sample size, age, gender, dose of radiation, and gross total [GTR] or near total [NTR] at time of radiation), in addition to the outcomes (event and total occurrence of symptomatic brainstem toxicity, grading of brainstem toxicity, and death from brainstem toxicity).

### 2.6. Statistical Analysis

A single-arm meta-analysis was performed using Open Meta Analyst, to calculate the pooled prevalence of the outcomes. We also conducted a subgroup analysis based on the grades of toxicity. This was conducted at 95% confidence intervals (CIs) using the untransformed proportion and random effects model.

## 3. Results

### 3.1. Searching and Screening

**Figure 1:**
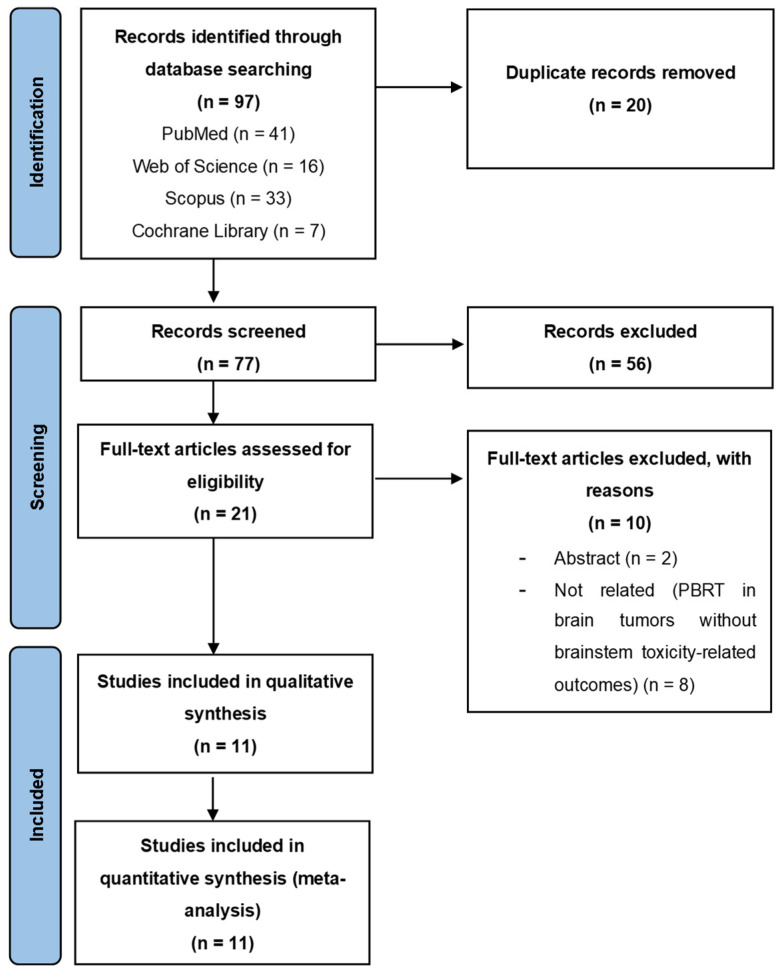
PRISMA flowchart of the literature search.

The literature search yielded a total of 97 articles. After removing the duplicates and screening the articles by title and abstract, 21 studies underwent the full text screening process. A total of 11 articles met the inclusion criteria and were included in our study [6,9,13,16,24,25,26,27,28,29,30]. The study selection process is displayed in a Preferred Reporting Items for Systematic Review and Meta-Analysis (PRISMA) flowchart (Figure 1).

### 3.2. Quality Assessment

NOS was used to assess the quality of the included articles. Five studies were judged to have moderate quality, whereas six studies had high quality (Table 1).

### 3.3. Baseline Characteristics

All 11 included studies were cohort studies. Nine were conducted in the USA, one in Switzerland, and one in the UK. The total sample size of patients was 2210, with most being male. The mean age ranged from 3.01 to 14.8 years old. The median radiation dose ranged from 54 to 59.4 Gy. The baseline characteristics are detailed in Table 2.

### 3.4. Statistical Analysis

#### 3.4.1. Brainstem Toxicity Incidence

**Figure 2:**
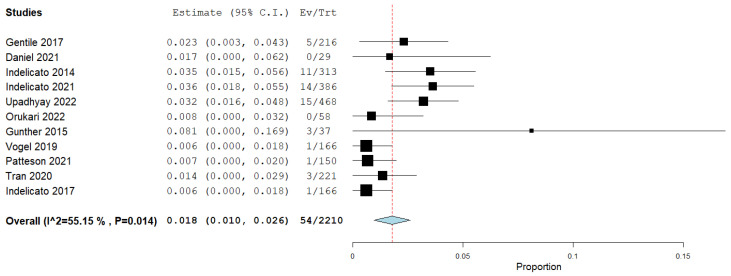
Forest plot showing the proportion of patients with brainstem toxicity [6,9,13,16,24,25,26,27,28,29,30].

A meta-analysis was conducted, which revealed that the pooled prevalence of patients who suffered from brainstem toxicity was 1.8% (95% CI: 1%, 2.6%) (Figure 2).

#### 3.4.2. Brainstem Toxicity Grades

**Figure 3:**
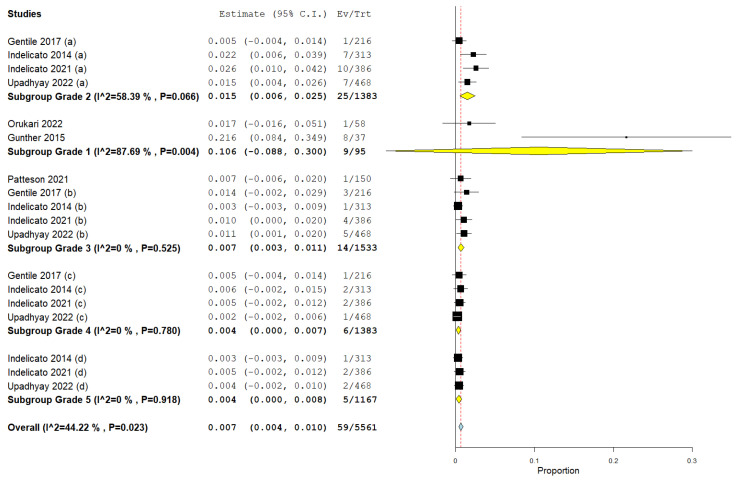
Forest plot showing the proportion of Grade 1, Grade 2, Grade 3, Grade 4, and Grade 5 brainstem toxicity [6,9,13,25,26,27,28].

A meta-analysis was conducted, which revealed that the pooled prevalences of patients with Grade 1 to Grade 5 brainstem toxicity were 10.6% (95% CI: 8.8%, 30%), 1.5% (95% CI: 0.6%, 2.5%), 0.7% (95% CI: 0.3%, 1.1%), 0.4% (95% CI: 0.1%, 0.7%), and 0.4% (95% CI: 0.1%, 0.8%), respectively, with an overall pooled prevalence of 0.7% (95% CI: 0.4%, 1%) (Figure 3).

#### 3.4.3. Death Following Brainstem Toxicity

A meta-analysis was conducted, which revealed that the pooled prevalence of death from brainstem toxicity was 0.5% (95% CI: 0.1%, 0.9%).

## 4. Discussion

### 4.1. Summary of Findings

The current study noted a small pooled prevalence of symptomatic brainstem toxicity occurring as a result of PBRT. Grade 1 was the most prevalent and is asymptomatic, whereas Grades 4 and 5 were the least common. Death from brainstem toxicity was also rare, with a pooled prevalence of only 0.5%.

### 4.2. Factors Affecting Brainstem Toxicity

The existing literature has identified several clinical variables that are related to an increased risk of symptomatic brainstem injury (SBI), including patient age and tumor site [9,18,31]. Upadhyay et al. [26] discovered a noteworthy correlation between SBI and the age and gender of patients. Out of the fifteen patients who experienced SBI, eight were three years old or younger when they underwent radiation therapy. Indelicato et al. demonstrated a correlation between individuals aged 5 years or less and the location of the tumor in the infratentorial region, which might explain the higher incidence of SBI in the younger population. It is worth mentioning that, according to their research, 52% of the tumors were supratentorial, whereas only 36% were infratentorial [9].

Upadhyay et al. [26] did not discover any correlation between SBI and pre-existing conditions during radiation therapy, such as posterior fossa syndrome, the extent and number of resections, and hydrocephalus. Curiously, out of the 15 patients who experienced SBI, 12 were female. Although the identification of female gender as a risk factor for SBI has been reported, other research institutes have not replicated this finding. Although this finding is significant, additional confirmation through further research is required to rule out the possibility of a false result.

At St. Jude, a higher incidence of incomplete brainstem function recovery at 12 months after radiotherapy was observed in boys compared to girls, indicating that males have a greater likelihood of experiencing brainstem injury [32]. Upadhyay et al. [26] observed that tumor type is a significant risk factor associated with SBI.

The treatment approach for brain tumors is determined by the specific type of tumor. For instance, patients with atypical teratoid rhabdoid tumors (ATRTs) usually undergo intense chemotherapy along with stem cell rescue. On the other hand, ependymoma patients are frequently prescribed greater doses of radiation. This analysis elucidates the association between the risk of SBI and the specific type of tumor, revealing a significantly higher risk in ATRT histology (11.6%) and ependymoma (5.8%), whereas medulloblastoma has a comparatively lower risk (1%) [26].

This has been corroborated by prior research [13,14]. Giantsoudi et al. [33] showed lower rates of SBI, specifically in medulloblastomas. Similarly, Gentile et al. [6] included a patient population with 71% medulloblastomas and only 3% ATRT histology, and also reported lower rates of SBI. The increased likelihood of developing SBI in individuals who do not undergo craniospinal irradiation (CSI) may be attributed to the fact that most medulloblastoma patients receive CSI, which results in a lower total dosage of radiation in the posterior fossa, ranging from 54 to 55.8 Gy. Upadhyay et al. [26] also noted a tendency towards decreased risk of SBI in individuals who underwent cone-down boost fields, suggesting that using phased radiation treatment with smaller margins near the brainstem could potentially decrease brainstem toxicity.

### 4.3. Dosing Parameters

The limits for acceptable doses to the brainstem are not uniformly established and are based on diverse data from adults and children. The Quantitative Analysis of Normal Tissue Effects in the Clinic (QUANTEC) guidelines, published in 2010, suggest that the entire brainstem can tolerate a radiation dose of 54 Gy, whereas smaller volumes (1–10 cc) can tolerate up to 59 Gy (administered in fractions of less than 2.0 Gy), with a risk of severe brainstem toxicity of less than 5% [34]. The latest Pediatric Normal Tissue Effects in the Clinic (PENTEC) guidelines propose that children have a 5% chance of developing necrosis when exposed to a radiation dose of 58.9 Gy, administered in fractions of 2 Gy each, to any region of the brain [35]. Upadhyay et al. [26] discovered that the V50–V52 values were notably greater in patients who experienced SBI in comparison to the remaining group. This was evident from the distinct divergence of the mean dose volume histogram (DVH) curves in the high-dose area. Murphy et al. [15] discovered a notable increase in V50, V52, and V54 among patients who received photon treatment and acquired necrosis. On the other hand, Gentile et al. [6] observed a reduced likelihood of SBI when using protons, as long as the maximum dose (Dmax) remained below 55.8 Gy RBE and the volume receiving 55 Gy RBE (V55) was kept at or below 6%.

### 4.4. Adverse Consequences Associated with Radiation Exposure

Tran et al. discovered a rate of 1.4% for severe brainstem radiation necrosis, consistent with the 1.3% rate reported by three prominent pediatric cancer centers that used proton radiation. This rate is also comparable to the 1.6–2.5% to 3.7% incidence observed in photon cohorts [15]. One approach to prevent brainstem radiation necrosis is implementing volumetric dose limitations [8]. The study conducted by Tran et al. found that the rate of late seizures in this cohort was 5.4%, which is lower than the data from the Childhood Cancer Survivor Study (CCSS) [36]. This difference in seizure rates may be attributed to the low rate of radiation necrosis, which was 30%.

The prevalence of moyamoya disease at the latest follow-up in this series, as reported by Tran et al., was 1.8%, which is half of the 3.5% reported previously [37]. Tran et al. also stated that cognitive impairment was observed in five instances, accounting for 2.3% of the total cases. The minimal rate of cognitive loss observed may be attributed to the inconsistent documentation of this measurement during the subsequent monitoring period. Olsson et al. [38] discovered a prevalence of 14% for mental retardation and/or overall diminished cognitive ability in children with brain tumors who underwent conventional radiation (CRT), based on objective measurements.

Prospective data were necessary to validate the potential enhanced cognitive results of proton therapy, as described by Gross et al. [39] and Kahalley et al. [40]. In the St. Jude Lifetime Cohort Study, it was found that 51.4% of children with brain tumors who had cranial CRT experienced long-term pituitary deficits [40]. Similarly, Shalitin et al. [41] reported a prevalence rate of 50%. Vatner et al. [42] discovered that young patients treated with protons had a hormone insufficiency rate of 55.5% over a period of 5 years. It is important to mention that there are five cases where irradiation in the hypothalamic region is believed to have caused endocrinopathy. These cases underscore the significance of being extremely cautious to prevent any harm to such tissues whenever feasible.

Tran et al. frequently reported other neurological illnesses, such as motor issues, ataxia, and cranial nerve disorders, which aligns with the results of the CCSS study [36]. The majority (80%) of abnormalities were attributed to local tumor invasion or surgical resection operations. Therefore, it is unlikely that the irradiation method will significantly enhance these outcomes. There is a tremendous need for protocols that not only aim to delay or reduce the intensity of radiation, but also optimize the effectiveness of all treatment methods [43,44].

The observed secondary metastasis rate of 1.4% reported by Tran et al. is encouraging. However, a longer follow-up period is necessary to accurately capture this occurrence, as it usually happens many years after treatment [45]. The early initiation of treatment at a young age and the presence of glial cells in the secondary metastasis are consistent with earlier research [46,47]. When analyzing the Pediatric Quality of Life (PEDQOL) data, distinct scoring patterns were noticed between proxy and self-assessments [29]. Contrary to the expectations of parents or caregivers, patients generally rated their quality of life (QoL) higher than the average; this is a widely recognized pattern in QoL publications [48,49,50].

The scores for cognition and social functioning were found to be significantly lower at later time periods compared to before proton therapy. This indicates the presence of common late-stage intellectual impairments and difficulties in social adaptation among individuals in this diagnostic group. This indicates that although proton treatment may have a lesser negative impact on patients compared to photons, it may not eliminate the possibility of late cognitive impairment. This impairment can be caused by multiple factors such as tumor location, surgery, radiation, chemotherapy, and patient-specific diseases.

Further strategies are required to avoid cognitive deterioration, encompassing various approaches such as hippocampus sparing [51,52]. Tran et al. reported that before proton therapy, Family Functioning and Global Well-Being were below average, but after 5 years of treatment, they reached values close to the average. In the most general sense, this suggests that any limits, if they exist, do not have a negative impact on the patient’s emotional well-being and ability to handle daily activities. Kuhlthau et al. [53] conducted a prospective assessment of health-related QoL in children with brain tumors treated with protons. The researchers discovered that the various self- and proxy-reported scores remained significantly associated with objective tests and exhibited a positive overall trend. Conversely, patients with CCSS reported lower levels of physical function, overall distress, and life satisfaction compared to their siblings [54]. Compared to data from the photon era, the favorable long-term QoL recorded in proton series, including the present study, indicates a potential advantage in preserving QoL.

### 4.5. Difference between Protons and Photons and Associated Factors

Robust dosimetric results indicate that PBRT can preserve more brainstem tissue compared to photon-based treatments [55]. Controversy persists over whether PBRT is linked to increased rates of brainstem toxicity (0–10.8%) compared to photons (0–6.7%) in pediatric posterior fossa brain tumors, culminating in a pivotal 2014 study that delineated its risk factors [9]. Indelicato et al. [9]. examined the cases of 313 pediatric patients treated at the University of Florida (UF) Health Proton Therapy Institute and identified factors linked to an elevated risk of brainstem toxicity, which included age below 5 years, tumor localization in the posterior fossa, median dose (D50%) exceeding 52.4 GyRBE, dose to 10% of the brainstem (D10%) surpassing 55.4 GyRBE, and Dmax exceeding 56.6 GyRBE. In light of these findings, revised guidelines were proposed, specifying target and maximum dosages [8]. According to the new recommendations, all patients must not exceed any maximum limits, and only one goal constraint may be exceeded. Additionally, for patients under 5 years of age, no goal constraint should be exceeded. The pediatric proton radiation oncology community has usually followed these limits or comparable, more cautious brainstem dosage limitations, as previously articulated by Haas-Kogan et al. [8] Additional single-institution studies assessing pediatric patients undergoing PBRT for posterior fossa cancers have corroborated more conservative dose limits than those previously allowed in the Children’s Oncology Group (COG) ependymoma protocols [8,9].

### 4.6. Emerging Trends in Proton Beam Radiation Thrapy

There are emerging trends that could potentially help improve the utility and outcomes of PBRT. FLASH radiotherapy has gained a lot of attention over the past few years as it can significantly reduce damage to the surrounding healthy tissues and its associated side effects through the delivery of ultra-high dose rates of radiation. However, using proton radiation in FLASH therapy in clinical practice is still under investigation, with increasing interest in it due to the superiority of proton beam radiation in targeting deep-seated tumors [56]. Pencil beam scanning is a modern technique used in proton radiation to enhance its precision in delivering radiation, with promising research results. Ares et al. [57] demonstrated favorable 5-year overall survival rates and local control rates of 84 ± 6.8% and 78 ± 7.5%, respectively, using pencil beam scanning proton therapy (PBSPT) in children diagnosed with intracranial ependymoma, despite the high-grade histology observed in 92% of patients. Leiser et al. [58] also found that using PBSPT in pediatric patients with rhabdomyosarcoma resulted in low toxicity rates, with QoL scores comparable to those of healthy individuals, and 5-year overall survival rates and 5-year local control survival rates of 80.6% and 78.5%, respectively. Adaptive radiation is another treatment approach that takes into account the changes that occur in patients’ anatomy, leading to more personalized treatment plans through modifications that address these changes [59].

### 4.7. Limitations and Recommendations

The current study is constrained by its design as a single-arm study, meaning it does not include a control or comparison group due to practical limitations. Furthermore, future studies on the use of proton treatment in children with brain tumors should specifically focus on addressing cognitive decline and QoL as crucial factors.

## 5. Conclusions

The present investigation documented a low proportion of symptomatic brainstem toxicity arising from PBRT. Grade 1 had the highest occurrence rate and was asymptomatic, whereas Grades 4 and 5 were the least frequent. Incidents of death caused by brainstem toxicity were infrequent, accounting for only 0.4% of the cases. As we mentioned, emerging trends suggest the use of advanced techniques in PBRT that could help improve its outcomes and toxicity profile, including the incidence of SBI. However, future studies are recommended to investigate many influencing factors, such as patient demographics, tumor type, and genetic predisposition, and their association with PBRT-induced SBI. In addition, exploring the effects of PBRT on different parameters related to the nervous system, other than brainstem toxicity, is also recommended to expand our knowledge about this treatment option and its implications. We also recommend establishing clear dosing guidelines that can be followed to potentially reduce PBRT-induced SBI.

## Figures and Tables

**Table 1:** Quality assessment of the included cohort studies using Newcastle–Ottawa Scale (NOS).

Study Name	Representativeness of the Exposed Cohort (★)	Selection of the Non-Exposed Cohort (★)	Ascertainment of Exposure (★)	Demonstration That Outcome of Interest Was Not Present at Start of Study (★)	Comparability of Cohorts on the Basis of the Design or Analysis (max★★)	Assessment of Outcome (★)	Was Follow Up Long Enough for Outcomes to Occur? (★)	Adequacy of Follow Up of Cohorts (★)	Quality Level
Gentile 2017 [6]	★	★	★	★	★★	★	0	★	High (8)
Indelicato 2021 [24]	★	★	0	★	★	★	★	0	Moderate (6)
Indelicato 2014 [9]	★	★	0	0	★★	0	★	★	Moderate (6)
Indelicato 2021 [25]	★	★	0	★	★	★	★	0	Moderate (6)
Upadhyay 2022 [26]	★	★	0	★	★	★	★	0	Moderate (6)
Orukari 2022 [27]	★	★	0	0	★★	★	★	★	High (7)
Gunther 2015 [13]	★	★	★	★	★★	0	0	★	High (7)
Vogel 2019 [16]	★	★	★	★	★★	★	0	0	High (7)
Patteson 2021 [28]	★	★	★	★	★★	★	★	0	High (8)
Tran 2020 [29]	★	★	★	★	★★	0	★	★	High (8)
Indelicato 2017 [30]	0	0	★	0	★★	★	★	★	Moderate (6)

★ indicates a degree for each question if the study aligned with the question and 0 indicates no degree if the study failed to align with the question.

**Table 2:** Baseline characteristics of the included studies.

Study ID	Location of Study	Study Design	Sample Size	Sex, Male n (%)	Age, Years Mean (SD)	Total Radiation Dose, GyRBE Median (IQR)	Gross Total (GTR) or Near Total (NTR) at Time of Radiation n (%)
Gentile 2017 [6]	USA	Cohort	216	126 (58.3%)	6.6 (3.77)	54 (46.8–59.4)	187 (86.6%)
Indelicato 2021 [24]	USA	Cohort	29	20 (69%)	14.8 (4.5)	NR	17 (59%)
Indelicato 2014 [9]	USA	Cohort	313	168 (53.7%)	5.9 (2.9)	NR	109 (34.8%)
Indelicato 2021 [25]	USA	Cohort	386	216 (55.9%)	3.8 (3.43)	55.8 (50.4–59.4)	328 (85%)
Upadhyay 2022 [26]	USA	Cohort	468	263 (56.2%)	6.25 (3.1)	54 (39.6–59.4)	288 (63.4%)
Orukari 2022 [27]	USA	Cohort	58	38 (65.5%)	10.3 (5.225)	54 (50.4–60)	52 (90%)
Gunther 2015 [13]	USA	Cohort	37	22 (59%)	3.01 (0.97)	59.4 (53.0–59.4)	37 (100%)
Vogel 2019 [16]	USA	Cohort	166	107 (64%)	10 (3.42)	54.0 (30.0–63.0)	100 (60%)
Patteson 2021 [28]	USA	Cohort	150	81 (54.0%)	3.6 (3.43)	54 (50.4–59.4)	121 (80.6%)
Tran 2020 [29]	Switzerland	Cohort	221	129 (58.4%)	4.1 (2.9)	54 (18–64.8)	79 (35.7%)
Indelicato 2017 [30]	UK	Cohort	166	90 (54%)	NR	NR	NR

SD: standard deviation, NR: not reported, IQR: interquartile range, RBE: relative biological effectiveness.

## Data Availability

All data are available on the internet.
